# A 63-Year-Old Man With a “Clicking Sound” in the Chest on Respiration

**DOI:** 10.1016/j.chest.2025.08.009

**Published:** 2025-12-09

**Authors:** Wytze S. de Boer, W.R. Douma, T.J. Olgers, Y.A. de Reus

**Affiliations:** a Department of Pulmonary Diseases, University of Groningen, University Medical Center Groningen, Groningen, the Netherlands; b Department of Internal Medicine, University of Groningen, University Medical Center Groningen, Groningen, the Netherlands

A 63-year-old man presented with a right-sided clicking sound in the chest during respiration, which had been present for 3 to 4 months. He described the sound as bothersome and distracting, persisting throughout the day and worsening in the upright position. He also reported increased fatigue and dyspnea, prompting his current visit. His medical history includes yellow nail syndrome (YNS) with associated chronic rhinosinusitis, bronchiectasis, and bilateral lymphocytic exudative pleural effusions. Intermittent pleural drainage had previously been effective, with the most recent procedure performed 11 months ago on the left side. On physical examination, auscultation revealed loud single or double “clicks” synchronized with respiration, audible throughout the right lung, and most prominently in the right axillary and anterolateral chest, just below the nipple line. The “clicks” were also perceptible on palpation and audible without a stethoscope. Chest radiography showed progressive accumulation of pleural effusion compared with imaging after the last drainage the previous year, with a small effusion on the right side, and prominent effusion on the left side. Thoracic ultrasonography (TUS) was performed to further evaluate the nature of the clicking sound (Videos 1, 2).

*Question: Based on the clinical scenario and TUS finding, what is the diagnosis?*

*Answer:* Pleural friction rub due to yellow nail syndrome with pleural effusion

TUS revealed abrupt, jerky movements of the pleural line in the right ventral chest, precisely at the location where the clicking sound was most prominent on auscultation. These shock-like pleural movements were synchronized with the audible clicks, strongly supporting the diagnosis of a pleural friction rub. This finding aligns with the clinical context of YNS, a condition known to cause recurrent pleural effusions and pleural inflammation, both of which can lead to pleural rubbing. No alternative diagnosis was observed during TUS, such as rib fractures or rib-tip syndrome.

## Discussion

A pleural friction rub is a coarse, grating sound produced when inflamed visceral and parietal pleura rub against each other during respiration.^1^ Typically the expiratory component mirrors the inspiratory component and may resemble crackles or even may mimic valvular cardiac sounds, complicating bedside interpretation.^2^ The pleural friction rub is probably produced by the sudden release of tangential energy from a lung surface that is temporarily prevented from sliding because of a frictional force between the 2 pleural layers.^2^

Although pleural rub is traditionally an auscultatory diagnosis, this case demonstrates that ultrasound can directly visualize the mechanical correlate. In this patient, thoracic ultrasonography showed jerky, shock-like movements of the pleura at the location of maximal auscultatory findings, synchronous with the audible clicks. This visual-audio correlation supports the diagnosis of a pleural friction rub.

YNS is a rare disorder characterized by the triad of yellow dystrophic nails, lymphatic dysfunction, and respiratory manifestations.^3^ The disorder is estimated to have a prevalence of less than 1 per 1,000,000^4^ and was first described by Samman and White in 1964.^5^

Although the pathogenesis of YNS remains incompletely understood, it is generally considered an acquired disorder with impaired lymphatic transport.^5^ Multiple possible causes have been reported—autoimmune diseases, malignancies, immunodeficiencies, major surgery, and exposure to titanium. It has also been reported in familial cases, suggesting a possible hereditary variant.^6^

Respiratory manifestations are common and diverse, affecting more than one-half of patients and may precede nail discoloration.^6^ Chronic cough is the most frequent symptom, followed by pleural effusions, which occur in 14% to 46% of cases and are typically bilateral, lymphocytic, and exudative, although sometimes chylous in nature (20%-30% of cases).^4^^,^^5^ These effusions tend to recur and may require management with thoracentesis, pleurodesis, or (in chylous effusions) thoracic duct ligation.^4^ Bronchiectasis is present in approximately 44% of patients, usually affecting the bilateral lower lobes and associated with recurrent lower respiratory infections.^4^^,^^5^ Chronic sinusitis is also reported in approximately 40% of cases.^5^ Results of pulmonary function tests may be normal or show restrictive patterns caused by effusions.^5^

Management of pleural effusions in YNS remains supportive and symptom-driven.^6^ Options include therapeutic thoracentesis, pleurodesis, or long-term indwelling catheters in recurrent cases. In some patients, treatment with octreotide, antifungals, or vitamin E has been explored, although evidence remains limited.^6^ In this case, the right-sided symptoms were managed conservatively, whereas therapeutic thoracentesis was arranged for the progressive left-sided effusion.

## Reverberations

1. *Pleural friction rubs can present as audible clicks and may be visualized using point-of-care ultrasound.*
2. *TUS can serve as a valuable adjunct to physical examination by directly correlating abnormal respiratory sounds with dynamic pleural surface findings.*
3. *In YNS, recurrent lymphocytic exudative pleural effusions are common and can contribute to chronic pleural inflammation and mechanical pleural rubbing.*

## Financial/Nonfinancial Disclosures

None declared.
